# Transcriptomic and Metabolomic Analyses Reveal Molecular Regulatory Networks for Pigmentation Deposition in Sheep

**DOI:** 10.3390/ijms25158248

**Published:** 2024-07-28

**Authors:** Mancheng Zhang, Xiaoli Xu, Yuan Chen, Chengqi Wei, Siyuan Zhan, Jiaxue Cao, Jiazhong Guo, Dinghui Dai, Linjie Wang, Tao Zhong, Hongping Zhang, Li Li

**Affiliations:** Farm Animal Genetic Resources Exploration Innovation Key Laboratory of Sichuan Province, College of Animal Science and Technology, Sichuan Agricultural University, Chengdu 611130, China

**Keywords:** melanin, transcriptome, metabolome, skin, tongue, sheep

## Abstract

Domestic animals have multiple phenotypes of skin and coat color, which arise from different genes and their products, such as proteins and metabolites responsible with melanin deposition. However, the complex regulatory network of melanin synthesis remains to be fully unraveled. Here, the skin and tongue tissues of Liangshan black sheep (black group) and Liangshan semi-fine-wool sheep (pink group) were collected, stained with hematoxylin–eosin (HE) and Masson–Fontana, and the transcriptomic and metabolomic data were further analyzed. We found a large deposit of melanin granules in the epidermis of the black skin and tongue. Transcriptome and metabolome analysis identified 744 differentially expressed genes (DEGs) and 443 differentially expressed metabolites (DEMs) between the pink and black groups. Gene ontology (GO) and Kyoto encyclopedia of genes and genomes (KEGG) enrichment analyses revealed the DEGs and DEMs were mainly enriched in the pathways of secondary metabolic processes, melanin biosynthesis processes, melanin metabolism processes, melanosome membranes, pigment granule membranes, melanosome, tyrosine metabolism, and melanogenesis. Notably, we revealed the gene *ENSARG00020006042* may be a family member of *YWHAs* and involved in regulating melanin deposition. Furthermore, several essential genes (*TYR*, *TYRP1*, *DCT*, *PMEL*, *MLANA*, *SLC45A2*) were significantly associated with metabolite prostaglandins and compounds involved in sheep pigmentation. These findings provide new evidence of the strong correlation between prostaglandins and related compounds and key genes that regulate sheep melanin synthesis, furthering our understanding of the regulatory mechanisms and molecular breeding of pigmentation in sheep.

## 1. Introduction

In recent years, research on sheep’s coat and skin color has attracted significant attention because coat color is a prominent visual characteristic of animals and an important economic trait of sheep wool fibers. Additionally, melanin has a strong antioxidant effect, and it is believed that consuming foods containing melanin is beneficial to people’s health, so black foods are highly sought after, which are closely associated with the presence and regulation of melanin [1,2]. Melanin plays various vital roles in organisms and is commonly present in animal skin, hair, and mucous membranes, protecting against ultraviolet radiation, combating oxidative stress, facilitating visual function, and participating in cellular signal transduction, among others [3].

Melanin synthesis is regulated by critical genes such as *ASIP*, *MC1R*, *TYR*, *TYRP1*, *DCT*, *SLC45A2*, *OCA2*, and *PMEL* in the transcriptional regulation of the skin or coat color phenotypes in sheep [4,5,6,7], goats [8,9,10,11], cattle [12,13], chickens [2], pigs [14,15], and quail [16]. Additionally, melanin synthesis is intricately regulated by a convergence of signaling pathways and metabolic pathways such as the MC1R/α-MSH signaling pathway [17], PI3K/Akt signaling pathway, MAPK signaling pathway, Wnt/β-catenin signaling pathway [18,19], and tyrosine metabolism [20]. Therefore, multiple genes and pathways are involved in melanin synthesis, requiring a systematic study to unravel the complex regulatory network.

Currently, domestic animal melanin-related genes and their regulations are investigated based on genome or transcriptome analysis [21,22,23,24,25,26]. Meanwhile, Multi-omics analysis integrates different levels of biological information and provides a more comprehensive understanding of biological systems. In recent years, the integrated study of the transcriptome and metabolome has been widely used in animal husbandry [27,28,29,30], which reveals the intrinsic connection between gene expression and metabolic small molecules, and effectively explains complex phenotypic traits.

Liangshan black sheep is a traditional livestock breed with a black coat, strong disease resistance, flexible movement, and good meat production performance. Liangshan semi-fine-wool sheep is a dual-use wool and meat breed with white coats. In this study, to investigate the molecular network underlying pigment deposition, Liangshan black sheep (black coat, black skin, black tongue, and black mucosa) and Liangshan semi-fine-wool sheep (white coat, pink skin, pink tongue, and pink mucosa) under the same feeding conditions at the Butuo County, Liangshan region of Sichuan, China, were used as the experimental subjects. The sheep’s abdominal skin, tongue, and lip tissues were sampled and stained with HE and Masson–Fontana. We carefully considered the effect of ultraviolet light on melanin deposition. Thus, we selected the abdominal skin and tongues for transcriptomics and metabolomics studies to reveal the melanin deposition process and related molecular regulatory networks.

## 2. Results

### 2.1. Histological Analysis of Sheep Skin, Tongue, and Lips

**Figure 1:**
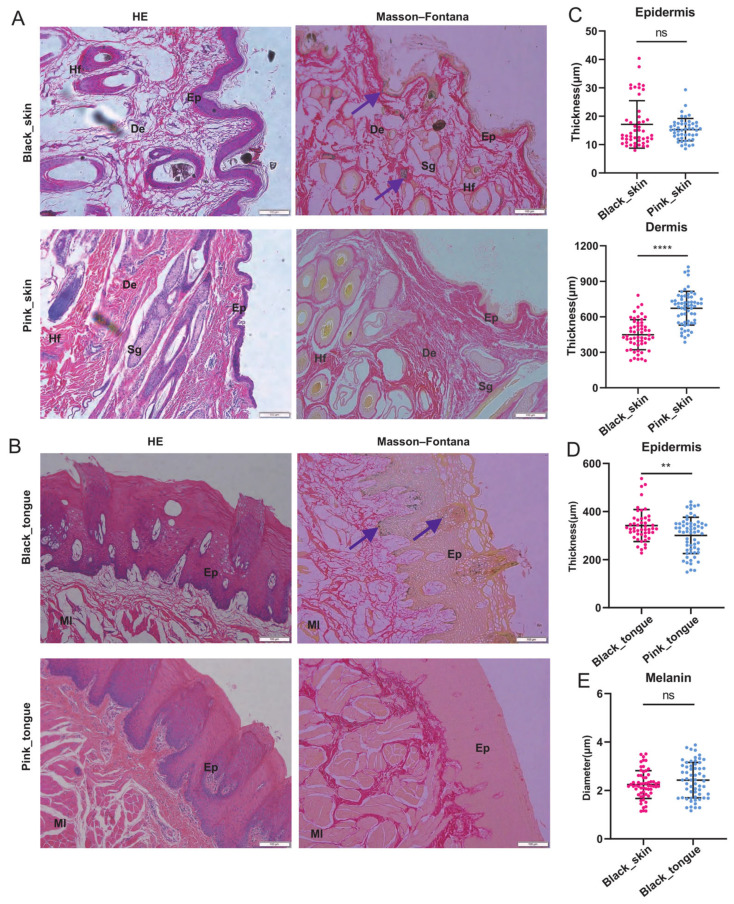
Morphological and melanin deposition characteristics of skin and tongue of sheep. (**A**) The skin stained with HE and Masson–Fontana stains. (**B**) The tongue stained with HE and Masson–Fontana stains. Scale bars: 100 μm. Ep: epidermal layer, De: dermal layer, Hf: hair follicle, Sg: sebaceous gland, Ml: muscular layer. Purple arrow: melanin. (**C**) Statistical analysis of skin epidermis and dermis. (**D**) Statistical analysis of tongue epidermis. (**E**) Black skin and black tongue melanin diameter size. Section count: 15 fields of view per section were selected for counting (*n* = 5). In the figure: “ns” indicates no significant difference, “**” indicates *p* < 0.01, “****” indicates *p* < 0.0001.

Compared to the white coat and pink tissues of Liangshan semi-fine-wool sheep, the coat, skin, and tongue of Liangshan black sheep were black and dark (Figure S1A), categorized into the pink and black groups, respectively. We found darker coloration of hair follicles and epithelial layers in the black skin compared to the pink skin, with a similar trend in the tongues and lips (Figure 1A,B and Figure S1B). The epidermis and dermis of the black lips were significantly thicker than those of the pink lips (Figure S1C). Similarly, the epidermis of the black tongues was also thicker than the pink ones (Figure 1D). However, the dermis of the pink skin was significantly thicker than that of the black skin (Figure 1C), and there was no significant difference in the epidermis thickness between the black and pink skin. Furthermore, Masson–Fontana staining showed many melanin granules diffused in the epidermis, hair follicles, skin, and tongue surface of the black sheep. However, there was no significant difference in the melanin particle diameter between the black skin and tongue (Figure 1E).

### 2.2. Screening Color-Related DEGs of Sheep Skin and Tongue

**Figure 2:**
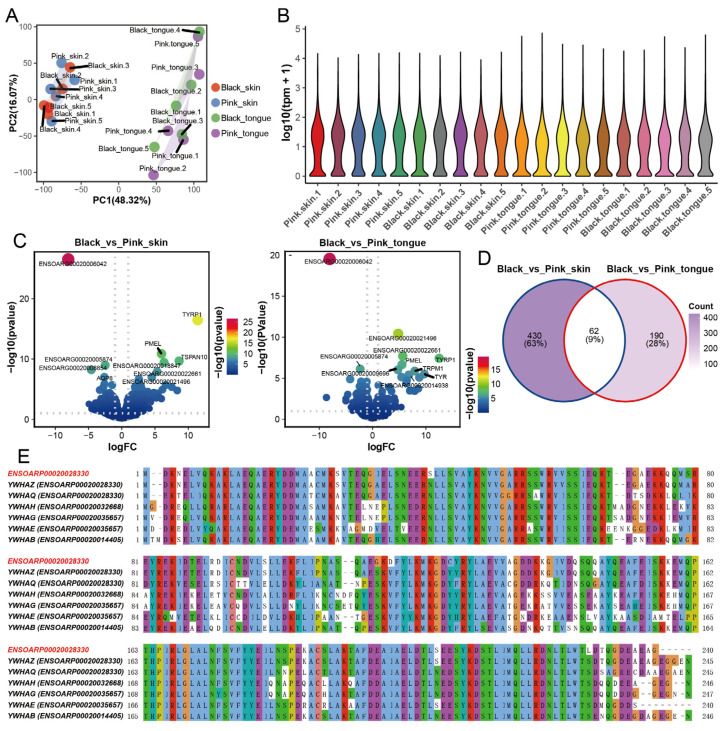
DEGs in skin and tongue between pink and black sheep. (**A**) PCA plot of 4 groups of samples. (**B**) Violin plot of gene expression in skin and tongue samples. (**C**) Volcanic map of DEGs in skin group and tongue group. (**D**) Venn diagram of DEGs in skin group and tongue group. (**E**) Amino acid sequence comparison between *ENSOARG00020006042* and *YWHAs* family. Note: *ENSOARP00020028330* is the protein ID of *ENSOARG00020006042*.

Using mRNA-seq, we obtained 848,560,516 clean reads from 20 sheep skin and tongue transcriptome data (Table S1). The average alignment rate of each sequencing sample to the reference genome was 95.73%. Principal component analysis (PCA) and correlation analysis showed that tissue was critical for categorizing gene expression, followed by sheep breed or color (Figure 2A and Figure S2A). Expectedly, there was no dramatic difference in the overall expression of genes across samples (Figure 2B). Additionally, we found 492 differentially expressed genes (DEGs) between the pink and black skin, among which 246 genes were upregulated and 246 downregulated (Table S2 and Figure 2C). Meanwhile, a total of 252 genes were differentially expressed in the black and pink tongues, with 148 genes upregulated and 104 genes downregulated (Table S3 and Figure 2C). The heatmap showed that the expression of genes altered in differently colored tissues (Figure S2B). There were 62 DEGs shared between the skin and tongues, including several typical genes involved in melanin deposition, such as *TYR*, *TYRP1*, *DCT*, *PMEL*, *SLC45A2*, and *MLANA* (Figure 2D), which is consistent with the previous studies [7,21,31]. To verify the results of mRNA-seq, we quantified the expression of six randomly selected genes through RT-qPCR. The consistent results indicate the high reliability of the mRNA-seq data (Figure S3).

Interestingly, we found the expression of the gene *ENSOARG00020006042* showed the most significant difference in both the skin and tongue groups (Figure 2C). A comparison of the amino acid sequence of *ENSOARG00020006042* revealed that it is conserved to the sheep *YWHAs* gene family protein, with the highest similarity with *YWHAZ* (Figure 2E). Furthermore, the *YWHAZ* protein is highly conserved across species (Figure S2C). *YWHAZ* has been identified as a connecting factor between tyrosinase-triggered melanin production and melanoma growth, highlighting its close association with melanin synthesis [32]. These results suggest that *ENSOARG00020006042* may function similarly to *YWHAZ* and is closely related to pigmentation regulation in sheep.

### 2.3. Function Enrichment of Color-Related DEGs of Sheep Skin and Tongue

**Figure 3:**
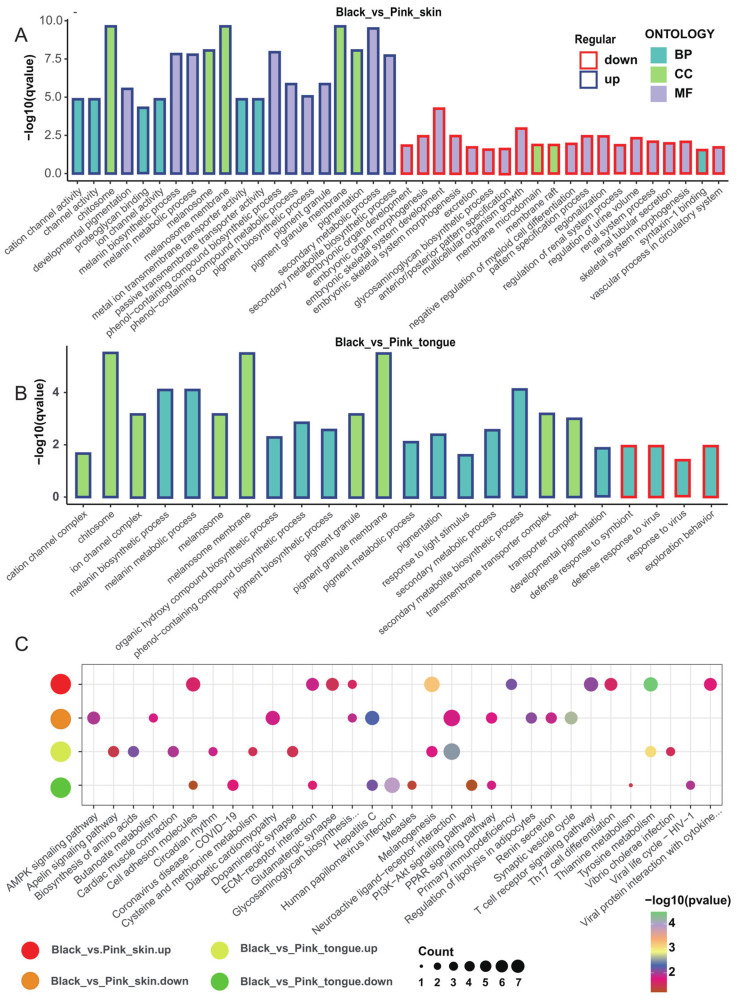
GO and KEGG enrichment analysis of DEGs associated with sheep pigmentation. (**A**) Top 20 GO enrichment terms for upregulated and downregulated genes in the skin. (**B**) Top 20 GO enrichment terms for upregulated and downregulated genes in the tongue. (**C**) Top 10 KEGG metabolic pathways for upregulated and downregulated genes in the skin and tongue tissues.

To explore the DEGs function, we performed GO and KEGG enrichment analysis. We revealed that compared with the pink animals, the DEGs upregulated in the black skin and tongue were significantly enriched in the secondary metabolite biosynthetic process, melanin biosynthesis process, melanin metabolism process, melanosome membranes, melanosome, and pigment granule membranes. Meanwhile, the downregulated DEGs in the black skin were enriched in embryonic skeletal system development and embryonic organ morphogenesis without relation to pigmentation (Figure 3A,B). Furthermore, we found 62 DEGs overlapped in the skin and tongue tissues and were concentrated on the melanin biosynthetic process, melanin metabolic process, pigment metabolic process, and tyrosine metabolic process (Figure S4A).

Moreover, DEGs upregulated in the black skin and tongue were significantly enriched in 13 and 12 KEGG metabolic pathways, including tyrosine metabolism and melanogenesis. In comparison, downregulated genes mainly functioned in 15 and 7 metabolic pathways, including the AMPK signaling pathway, PI3K−Akt signaling pathway, and PPAR signaling pathway (Figure 3C). Notably, those overlapping DEGs in the skin and tongue tissues were focused on tyrosine metabolism and melanogenesis (Figure S4B). Furthermore, the PPI network of the DEGs in the skin and tongue revealed that genes such as *TYR*, *TYRP1*, *DCT*, *SLC45A2*, *PMEL*, and *MLANA* were hub genes for sheep melanin synthesis (Figure S4C,D).

### 2.4. Color-Related Metabolites of Sheep Skin and Tongue

**Figure 4:**
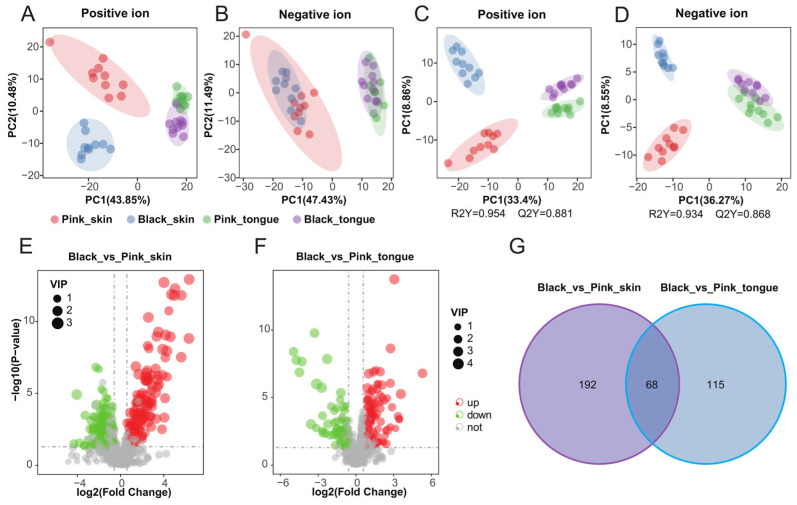
Pigmentation-related metabolomics of sheep skin and tongue. PCA maps of skin and tongue tissue samples in positive (**A**) and negative ion modes (**B**). PLS-da score plots for skin and tongue tissue samples in positive (**C**) and negative ion mode (**D**). Volcano plots of DEMs in the Black_vs_Pink_skin (**E**) and Black_vs_Pink_tongue (**F**) groups, respectively. (**G**) Venn diagram of DEMs in the Black_vs_Pink_skin and Black_vs_Pink_tongue groups.

To further anchor the molecules closely related to pigmentation, we performed metabolomics and screened 1486 metabolites on the sheep skin and tongue. Firstly, Pearson’s correlation analysis of the quality control (QC) samples in both positive and negative ion modes showed that the correlation coefficients between the QC samples were greater than 0.98 (Figure S5A,B), indicating that the data from our experimental samples are reliable. The principal component analysis (PCA) analysis showed differences between the groups (Figure 4A,B). There was an apparent classification between all groups in the positive ion mode. In contrast, in the negative ion mode, there was an obvious classification only between the different tissue samples, e.g., skin vs. tongue. We performed partial least squares discriminant analysis (PLS-da) on the data to further explore the differences between groups. The PLS-DA model effectively distinguished the pink and black skin and tongue tissues in positive and negative ion modes, indicating that the model was stable and reliable (Figure 4C,D).

Our research has identified 260 DEMs between the pink and black skin groups, with 149 upregulated and 111 downregulated (Figure 4E and Table S4). Similarly, 183 DEMs were found between the pink and black tongue groups, with 111 upregulated and 72 downregulated (Figure 4F and Table S5). Moreover, we discovered 68 common DEMs between the skin and tongue (Figure 4G). The clustered heat maps vividly showed the differences in metabolite concentrations in tissues of different colors (Figure S5C,D). Those results suggested that DEMs in the skin and tongue groups could significantly affect sheep’s pigment regulation.

### 2.5. KEGG Enrichment of Pigmentation-Related DEMs of Sheep Skin and Tongue

**Figure 5:**
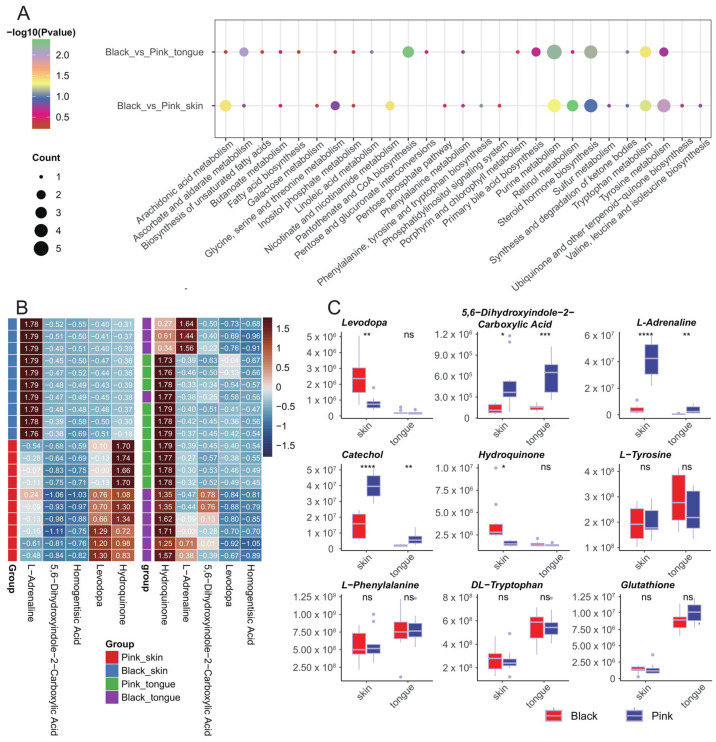
KEGG functional enrichment analysis of DEMs. (**A**) Enrichment bubble plots of KEGG metabolic pathways for DEMs. (**B**) Heatmap of metabolites enriched in the tyrosine metabolic pathway across samples. (**C**) Boxplots of the content of several metabolites in different tissues. In the figure: “ns” indicates no significant difference, “*” indicates *p* < 0.05, “**” indicates *p* < 0.01, “***” indicates *p* < 0.001, “****” indicates *p* < 0.0001.

The KEGG analysis of the DEMs in the skin group revealed five significantly enriched metabolic pathways: retinol metabolism, tyrosine metabolism, nicotinate and nicotinamide metabolism, arachidonic acid metabolism, and purine metabolism. Additionally, DEMs in the tongue group were enriched in four significant KEGG pathways, including pantothenate and CoA biosynthesis, purine metabolism, ascorbate and aldarate metabolism, and tryptophan metabolism (Figure 5A). We further analyzed the expression patterns of metabolites enriched in the tyrosine metabolic pathway in the skin and tongue. The results showed that 5,6-dihydroxyindole-2-carboxylic Acid and L-adrenaline significantly differed in both the skin and tongue, whereas levodopa and hydroquinone only differed in the skin. In addition, we valued the levels of several melanin-related metabolites, including L-tyrosine, catechol, L-phenylalanine, DL-tryptophan, and glutathione, of which only catechol presented significant differences between the black and pink skin as well as the tongue (Figure 5B,C).

In summary, the above metabolic pathways may be closely related to pigment regulation in sheep. In addition, in the tyrosine metabolic pathway, some intermediates’ concentration in the black synthesis process significantly differed between the black and pink groups. In contrast, the tyrosine concentration, the precursor substance of melanin synthesis, did not significantly differ between the black and pink groups.

### 2.6. Integrated Analysis of Pigmentation-Related Transcriptome and Metabolome of Sheep Skin and Tongue

**Figure 6:**
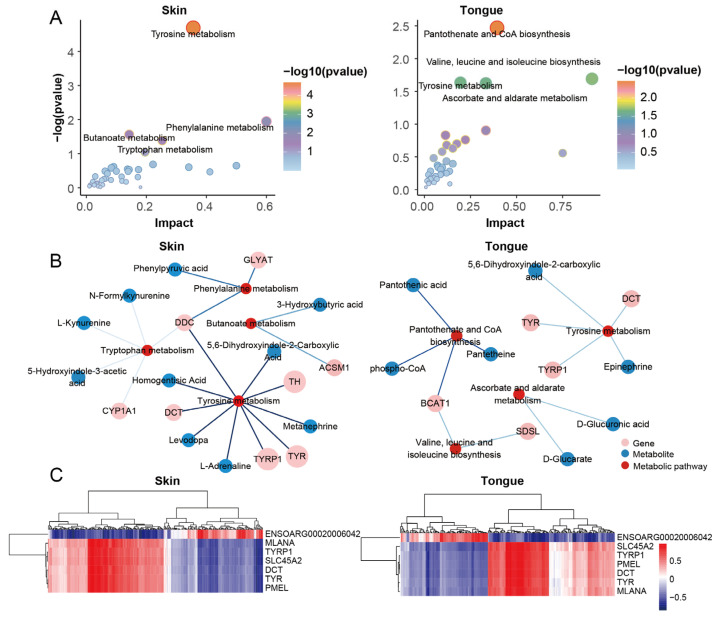
Integrative analysis for transcriptome and metabolome. (**A**) Joint pathway analysis of DEGs and DEMs in skin group and tongue group. (**B**) Network diagram of genes, metabolites, and metabolic pathways in the skin and tongue groups. (**C**) Heatmaps of genes and metabolites correlate in the skin group and tongue group.

We performed a joint pathway analysis of the DEGs and DEMs to further explore the relationship between genes and metabolites. We found that the tyrosine metabolism, phenylalanine metabolism, butyric acid ester metabolism, and tryptophan metabolism significantly functioned in the skin group. Meanwhile, the tongue group significantly enriched the pantothenate and CoA biosynthesis, valine, leucine, and isoleucine biosynthesis, tyrosine metabolism, ascorbate, and alternate metabolism. Notably, the tyrosine metabolism emerged in the skin and tongue (Figure 6A,B). These reinforce the importance of tyrosine metabolism in pigmentation in sheep.

**Figure 7:**
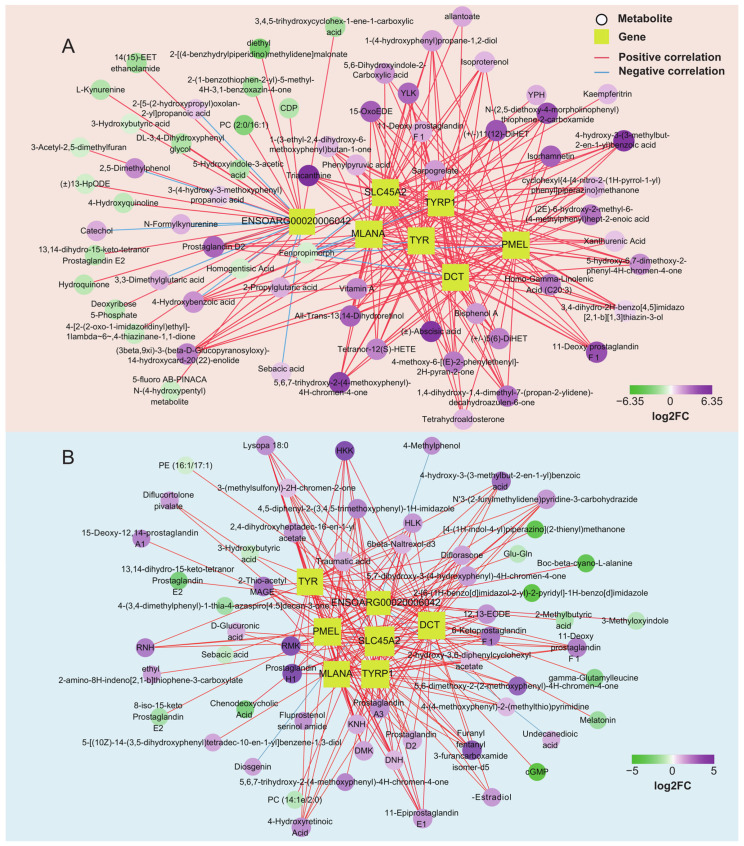
Network of pigmentation-associated genes and metabolites in the skin (**A**) and tongue group (**B**). (|r| > 0.75, *p* < 0.05).

Transcriptome analysis showed that the genes *TYR*, *TYRP1*, *DCT*, *PMEL*, *MLANA*, *SLC45A2*, and *ENSOARG00020006042* were the critical genes for pigmentation in sheep. We further analyzed the correlation between the above genes and differential metabolites in the skin and tongue groups (Figure 6C and Table S7). The results showed that 66 and 58 strongly correlated metabolites were screened in the skin and tongue groups, respectively (|r|> 0.75, *p* < 0.05) (Figure 7A,B). In the skin, there was a positive correlation between the gene *ENSOARG00020006042* and hydroquinone, 5-Hydroxyindole-3-acetic acid, 4-Hydroxyquinoline, and 3-hydroxybutyric acid. Also, this gene was negatively correlated with prostaglandin D2, catechol, and 3,3-dimethylglutaric acid. Moreover, there was a strong positive correlation between the genes *TYR*, *TYRP1*, *DCT*, *MLANA*, *PMEL,* and *SLC45A2* and the metabolites prostaglandin D2, 11-Deoxy prostaglandin F1β and 11-Deoxy prostaglandin F1α, isoproterenol, and 4-Hydroxybenzoic acid. Furthermore, the metabolite 5,6-dihydroxyindole-2-carboxylic acid was positively correlated with the expression of *TYRP1*, *PMEL*, and *SLC45A2*. In the tongue, *ENSOARG00020006042* was positively correlated with melatonin, cGMP, 8-iso-15-keto prostaglandin E2, and 3-hydroxybutyric acid but negatively linked to 4-methylphenol, diosgenin, and undecanedioic acid. There was a strong positive correlation between genes *TYRP1*, *SLC45A2*, *DCT*, and α-Estradiol. Additionally, *TYR*, *TYRP1*, *DCT*, *MLANA*, *PMEL*, and *SLC45A2* significantly correlated with the metabolites 6-Ketoprostaglandin F1α, prostaglandin A3, prostaglandin H1, prostaglandin D2, and 4-Hydroxyretinoic acid. These strong correlations between prostaglandin-related compounds and key genes regulating melanin synthesis demonstrate a close relationship between prostaglandins and melanin synthesis.

## 3. Discussion

Melanin is a pigment produced by cells that imparts color to animals’ hair, skin, and eyes [33]. It has been established that melanin possesses various functions, including the absorption of ultraviolet radiation, reduction in damage to skin cells caused by UV rays, scavenging free radicals, and regulation of photosensitivity [34]. The different coat and skin colors also determine the economic value of sheep’s wool and meat quality. To investigate the regulatory network of melanin deposition in sheep, we conducted transcriptomic and metabolomic analyses, comparing the differences in genes and metabolites between the color-related (pink and black) skin and tongue in sheep.

Melanin synthesis is a part of the tyrosine metabolism that primarily occurs in specialized organelles called melanosomes. In mammals, melanin consists of two chemical forms: eumelanin and pheomelanin [35]. The former is a brown-black or dark insoluble polymer, while the latter is a red-yellow soluble polymer [36]. *TYR* plays a crucial role in the process of melanin synthesis, participating in the production of both forms of melanin, and is considered the rate-limiting stage in the synthesis [37,38,39]. The synthesis of eumelanin also requires the catalytic action of *TYRP1* and *DCT* [40]. In contrast, pheomelanin synthesis is believed to occur autonomously but relies on non-enzymatic reactions involving cysteine or glutathione [41]. When *TYR* is expressed at higher levels, both forms of melanin increase, but when *TYRP1* and *TYRP2* are expressed at higher levels, only eumelanin synthesis is enhanced [42]. In this study, GO enrichment analysis revealed several pathways associated with melanin metabolism, including melanin biosynthetic, melanin metabolic, pigment deposition, and developmental pigmentation. The genes involved in these pathways include *TYR*, *TYRP1*, *DCT*, *PMEL*, *MLANA*, *SLC45A2*, *OCA2*, *MC1R*, and *KIT*. These findings are consistent with previous research results [6,7,31,43].

Interestingly, the expression pattern of the gene *ENSOARG00020006042* was opposite to that of the *TYR* genes family and had large-fold changes between the black and pink skin or tongue. The gene *ENSOARG00020006042* is located on the seventh chromosome of sheep, and its function has not yet been annotated. We found the *ENSOARG00020006042* exhibited the highest similarity with the *YWHAZ* gene, with a comparison rate of 96%, which indicates it is potentially a member of the *YWHA* gene family that is associated with melanogenesis and melanoma [32,44]. Moreover, the gene *YWHAZ* protein sequence is highly conserved across species. These suggest that this gene may play an essential role in melanin regulation.

DEGs and DEMs between the black and pink skin or tongue were enriched in the tyrosine metabolic pathway, and the common metabolites included L-adrenaline and 5,6-dihydroxyindole-2-carboxylic acid. The 5,6-dihydroxyindole-2-carboxylic acid is an intermediate in melanin synthesis [45,46,47]. Adrenaline can promote melanogenesis through the cAMP/PKA pathway [48,49]. In addition, adrenaline binds to α1 and β2 adrenergic receptors on melanocytes and activates the inositol triphosphate/diacylglycerol (IP3/DAG) pathway [50,51]. This activation leads to elevated intracellular PKC-β levels and the activation of tyrosinase, which regulates melanin synthesis. Among the results of the gene and metabolite correlation analyses, strong correlations were found between prostaglandins and related compounds and key genes regulating melanin synthesis. Prostaglandins (PGs) are lipid hormones produced by various cells and play multiple roles at the cellular and tissue level, such as cell development, angiogenesis, wound healing, and inflammatory responses. In the skin, various factors (including UV irradiation, cytokines, and trauma) stimulate the production of PGs by gliogenic cells, primarily PGE2 and PGF2α. PGE2 has four receptors, EP1, EP2, EP3, and EP4, while PGF2α has one, the FP receptor. Studies have found that melanocytes express EP1, EP3, and EP receptors. PGE2 and PGF2α act as paracrine factors that stimulate dendrite formation in melanocytes by interacting with G protein-coupled receptors [51,52] and regulating melanin deposition by modulating *TYR* levels. Previously, estrogen receptors were present in human melanocytes [53,54], and estrogen promotes the translocation of melanocytes to keratin-forming cells, leading to melanin deposition [55]. Our study showed a strong positive correlation between α-Estradiol and genes TYRP1, SLC45A2, and *DCT*. Moreover, there is a strong correlation between key genes for melanin synthesis, prostaglandins, and related compounds. Therefore, there may be a link between melanin deposition and sheep reproduction, but further studies are still needed to confirm this.

Climate change due to global warming leads to a rise in temperature and UV radiation. Individual animals of different colors have different sensitivities to climate change, with darker colors providing UV protection. Therefore, if global warming means an increase in solar radiation, dark-colored individuals may be less affected in the habitats of light-colored individuals. The gene for melanin deposition confers resistance to many stressors in individual animals. Melanin may be a major component of adaptation to warming, and thus, melanin-based skin is likely to change in animal populations as an evolutionary response to warming [56]. Compared with Liangshan semi-fine-wool sheep, Liangshan black sheep have black wool fibers and black skin phenotypes, so they are more suitable for the rough highland grazing environment.

Here, we systematically analyzed the molecular regulatory network of pigmentation in sheep. Genes such as *TYR*, *TYRP1*, *DCT*, *PMEL*, *MLANA*, and *SLC45A2* are collectively involved in pigmentation in sheep. In addition, the gene *ENSOARG00020006042* may be equally important in regulating pigmentation in sheep. DEGs and DEMs were significantly enriched in melanin biosynthesis, melanin metabolism processes, pigment granule membranes, melanosome, tyrosine metabolism, and melanogenesis. We also found strong correlations between prostaglandins and related compounds and estrogens and key genes regulating melanin synthesis, providing evidence for a link between melanin synthesis and reproduction in sheep and further deepening our understanding of the regulatory mechanisms and molecular breeding of pigmentation in sheep.

## 4. Materials and Methods

### 4.1. Animal and Sample Collection

The Liangshan black sheep and Liangshan semi-fine-wool sheep were from the original breeding farm of Liangshan black sheep in Butuo County, Liangshan Yi Autonomous Prefecture, Sichuan Province, and the original breeding farm of Liangshan semi-fine-wool sheep in Butuo County, Sichuan Province, respectively. All animals were fed hay and corn silage and had free access to water and mineral salts.

One-year-old *Liangshan black sheep* (with black wool, black skin, and black tongue, *n* = 10) and *Liangshan half-fine-wool sheep* (with white fur, pink skin, and pink tongue, *n* = 10) were selected as research subjects in a gender ratio of 1:1 (male: female). After 24 h fasting, the sheep were humanely sacrificed, and skin (abdominal region), tongue, and lip tissue samples were quickly collected. One set of samples was placed in liquid nitrogen and stored at −80 °C for further use. Another was fixed in a 4% formaldehyde solution prepared for the tissue section.

### 4.2. Skin and Tongue Tissue Section Preparation

The collected skin, tongue, and lip tissues were fixed in 4% paraformaldehyde (Servicebio, Wuhan, China) and stored in a refrigerator at 4 °C for 24 h. The fixed samples were rinsed and placed in a 70% ethanol solution (Sinopharm Group Co., Ltd., Shanghai, China) for 5–10 min to remove moisture. Dehydrated samples were soaked in a 1:1 mixture of absolute ethanol (Sinopharm Group Co., Ltd., Shanghai, China) and xylene (Sinopharm Group Co., Ltd., Shanghai, China) for 30 min and placed in xylene for 1 h before embedding in paraffin.

HE staining: The paraffin-embedded tissue blocks were cut into 5–7 μm thick sections using a microtome and mounted on glass slides (Servicebio, Wuhan, China). The slides were deparaffinized in xylene, and residual xylene was removed using alcohol. The sections were stained with HE (Servicebio, Wuhan, China) and dehydrated using a gradient of ethanol concentrations. Finally, the slices were sealed using neutral gum (Sinopharm Group Co., Ltd., Shanghai, China) and observed and photographed using a light microscope (Nikon Eclipse E100, Tokyo, Japan) and under an imaging system (Nikon DS-U3, Tokyo, Japan). The epidermis and dermis thickness were counted using ImageJ software v1.8.0 (https://imagej.net/ij/, accessed on 1 June 2023).

Masson–Fontana staining: Masson–Fontana staining is a specific method for staining melanin particles. The paraffin-embedded tissue blocks were cut into thin sections and deparaffinized with xylene. The sections were immersed in the melanin working solution and covered, then incubated in the dark at 4 °C for 12–18 h. After rinsing with distilled water three times, the sections were re-stained with the melanin staining solution (Servicebio, Wuhan, China). Finally, the sections were dehydrated with anhydrous ethanol, encapsulated with neutral resin, and observed and photographed under a light microscope (Nikon Eclipse E100, Tokyo, Japan) and imaging system (Nikon DS-U3, Tokyo, Japan). ImageJ software v1.8.0 was used for image analysis.

### 4.3. Transcriptome Sequencing and Metabolite Determination

#### 4.3.1. Transcriptome Sequencing

Transcriptome sequencing of skin and tongue tissues of male Liangshan black sheep (*n* = 5) and Liangshan semi-fine-wool sheep (*n* = 5) was performed. Total RNA was extracted from the skin and tongue tissues using the Trizol method. The integrity and quantity of RNA were assessed using the Agilent 2100 Bioanalyzer (Agilent Technologies, Palo Alto, Calif., USA). Qualified RNA samples were subjected to mRNA enrichment with poly(A) tails using Oligo(dT) magnetic beads, generating libraries. Finally, high-throughput sequencing was performed on the Illumina Novaseq 6000 (San Diego, California, USA) platform, generating paired-end 300 bp (2 × 150 bp) reads with a clean read depth of 6G for each library.

#### 4.3.2. LC-MS Non-Targeted Metabolome Assay of Sheep Skin and Tongue Tissues

The skin and tongue samples weighing 100 mg were finely ground using liquid nitrogen and transferred into a 1.5 mL EP tube. 500 μL of an 80% methanol–water solution was added. The mixture was thoroughly mixed using vortexing and incubated in an ice bath for 5 min. Subsequently, it was centrifuged at 15,000× *g* and 4 °C for 20 min. A specific volume (5 μL) of the resulting supernatant was taken and further diluted with mass spectrometry-grade water to attain a methanol content of 53%. The diluted sample was subjected to another centrifugation step at 15,000× *g* and 4 °C for 20 min, and the supernatant was collected. To create a quality control (QC) sample, an equal volume of the supernatant from each experimental sample was mixed. Both the experimental samples and the QC sample were analyzed using LC-MS.

The samples were subjected to separation using a Vanquish UHPLC system (Thermo Fisher, Karlsruhe, Germany) equipped with a Hypesil Gold column (Thermo Fisher, Karlsruhe, USA). The column temperature was maintained at 40 °C, and the flow rate was set at 0.2 mL/min. In the positive mode, mobile phase A consisted of 0.1% formic acid, while mobile phase B was methanol. For the negative mode, mobile phase A was 5 mM ammonium acetate (pH 9.0), and mobile phase B remained as methanol. The samples were analyzed using a Q Exactive™ HF mass spectrometer (Thermo Fisher, Karlsruhe, Germany) to acquire first- and second-order spectra. The scan range was set from *m*/*z* 100 to 1500. The ESI source settings included a spray voltage of 3.5 kV, sheath gas flow rate of 35 psi, auxiliary gas flow rate of 10 L/min, capillary temperature of 320 °C, S-lens RF level of 60, and auxiliary gas heater temperature of 350 °C. The polarity was adjusted to either positive or negative mode accordingly. MS/MS second-order scans were carried out using data-dependent acquisition.

### 4.4. Transcriptome and Metabolome Data Analysis

**Figure 8:**
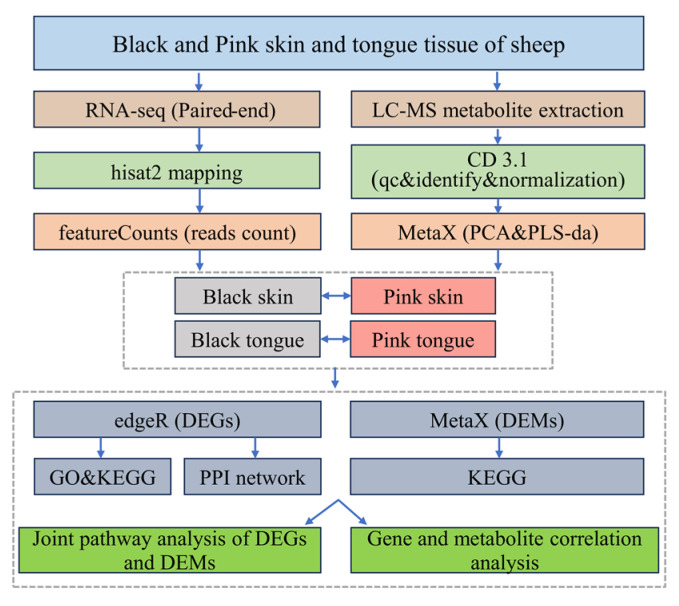
Data analysis flowchart.

Schematic of the transcriptomic and metabolomic data analysis process (Figure 8).

#### 4.4.1. Read Alignment and Gene Expression Calculation

Trimming: The raw data were evaluated using the software fastqc v0.11.9, followed by adapter and low-quality read trimming using fastp v0.23.2. The trimmed data were then re-evaluated using fastqc.

Alignment: The clean data were aligned to the Ensembl sheep reference genome Oar_rambouillet_v1.0 (release-108) using the software Hisat2 v2.1.0. The built-in Python scripts, extract_splice_sites.py and extract_exons.py, were used to extract splice site and exon coordinates from the reference genome annotation file (GTF, release-108). The index files were built using the hisat2-build parameters, and the data were aligned to the sheep reference genome using hisat2-align-s.

Bam Processing: the alignment results in SAM format were converted to BAM format, sorted, and processed using samtools v1.16.1.

Gene Expression Computation: The software featureCounts v2.0.1 was used to quantify gene expression by counting reads. Differential analysis was performed using the R (v4.2.3) package edgeR v3.40.2.

#### 4.4.2. Protein Interaction Network Analysis

The DEGs were subjected to gene–protein interaction network analysis using the STRING online platform (https://string-db.org accessed on 1 October 2023). The results were visualized using Cytoscape v3.10.0.

#### 4.4.3. Conservativeness Analysis of Protein Sequences

The protein sequence of *ENSOARG00020006042* was aligned using the blastP tool of the Ensembl database (https://www.ensembl.org/index.html, accessed on 12 November 2023) and visualized using the software Jalview v2.11.3.2.

The raw data files were imported into the CD v3.1 software for processing. Each metabolite was screened based on parameters such as retention time and mass-to-charge ratio. Peak alignment was performed using a retention time deviation of 0.2 min and a mass deviation of 5 ppm to improve identification accuracy. Peak extraction was then performed using parameters such as a mass deviation of 5 ppm, signal intensity deviation of 30%, signal-to-noise ratio of 3, minimum signal intensity, and summing ions. Peak areas were quantified, and target ions were integrated. Molecular formulas were predicted based on molecular ion peaks and fragment ions, and comparisons were made with the mzCloud, mzVault, and Masslist databases. Background ions were removed using blank samples, and the original quantitative results were normalized to obtain metabolite identification and relative quantification results.

#### 4.4.4. Multivariate Statistics and Metabolite Differential Analysis

Metabolite relative quantification data were log2 transformed using MetaX software v.1.4.16 [57], followed by PCA and PLS_da analyses using PCA function centering and z-value correction. VIP values were obtained for each metabolite. For univariate analysis, t-tests were used to calculate the statistical significance (*p*-value) and fold change (FC) for each metabolite between the two groups. The default criteria for differential metabolite selection were VIP > 1, *p*-value < 0.05, and FC ≥ 2 or FC ≤ 0.5.

#### 4.4.5. Functional Enrichment Analysis of DEGs and DEMs

The R packages org.Hs.eg.db v3.16.0 and clusterProfiler v4.6.2 performed GO and KEGG enrichment analysis on the DEGs. Human databases were used instead of incomplete sheep databases in this analysis.

KEGG enrichment analysis of DEMs and gene–metabolite joint pathway analysis was performed using an online website (https://www.metaboanalyst.ca, accessed 6 October 2023).

#### 4.4.6. Gene–Metabolite Correlation Analysis

Pearson correlation coefficient analysis between genes and metabolites was performed using the cor function in R. The statistical significance was determined using the cor.mtest function in R, with a *p*-value < 0.05 considered statistically significant.

### 4.5. Quantitative Real-Time PCR (qPCR)

The cDNA of mRNA was obtained by Primer Script^TM^ RT kit (Takara, Beijing, China). We normalized the gene expression levels using the reference gene *GAPDH* and calculated the relative RNA levels of genes using the 2^−ΔΔCt^ method. All primers are detailed in Table S6. All data are expressed as SEM ± mean (*n* = 4). The statistical significance of the data was analyzed using the t-test in the software GraphPad Prism v8.0.

## Data Availability

The raw sequencing data can be found online (https://www.cncb.ac.cn/, accessed on 1 January 2024, PRJCA024420).

## Supplementary Materials

The following supporting information can be downloaded at: https://www.mdpi.com/article/10.3390/ijms25158248/s1.
