# Supplementary material for: Transcriptomic and Metabolomic Analyses Reveal Molecular Regulatory Networks for Pigmentation Deposition in Sheep
Source: Int J Mol Sci. 2024 Jul 28;25(15):8248. doi: 10.3390/ijms25158248 (PMC11311981; doi:10.3390/ijms25158248)
Supplement: Supplementary file 1 [file ijms-25-08248-s001.zip › ijms-3098969-supplementary.pdf]

Supplementary material

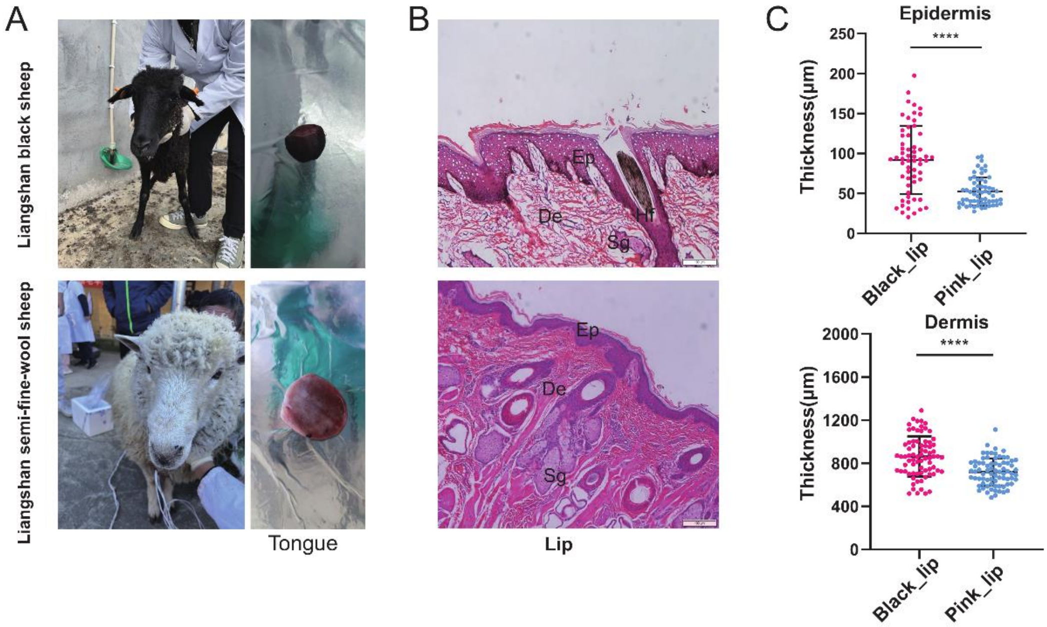

Figure S1. Comparison of characteristics between Liangshan black sheep and Liangshan semi-fine wool sheep. (A) Comparison of appearance and tongue characteristics between Liangshan black sheep and Liangshan semi-fine wool sheep. (B) HE-stained sections of sheep lips. Scale bars: 100μm. (C) Statistics of lip epidermis and dermal thickness.

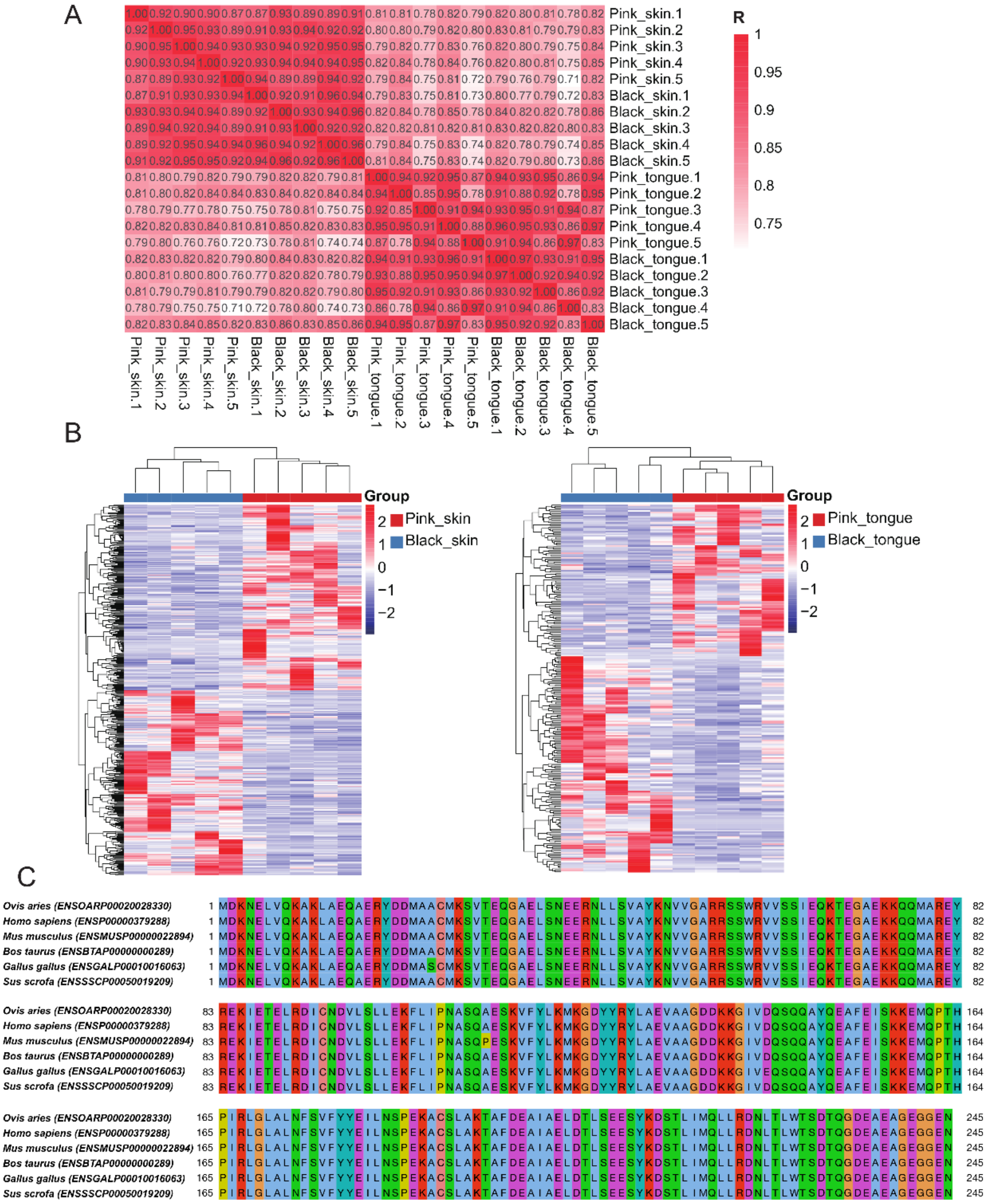

Figure S2. Differentially expressed genes analysis. (A) Heatmap of correlations across mRNA-seq samples. (B) Heatmap of differential genes between Black_vs_Pink_skin and Black_vs_Pink_tongue groups. (C) Conservation analysis of the protein sequence of the *YWHAZ* gene.

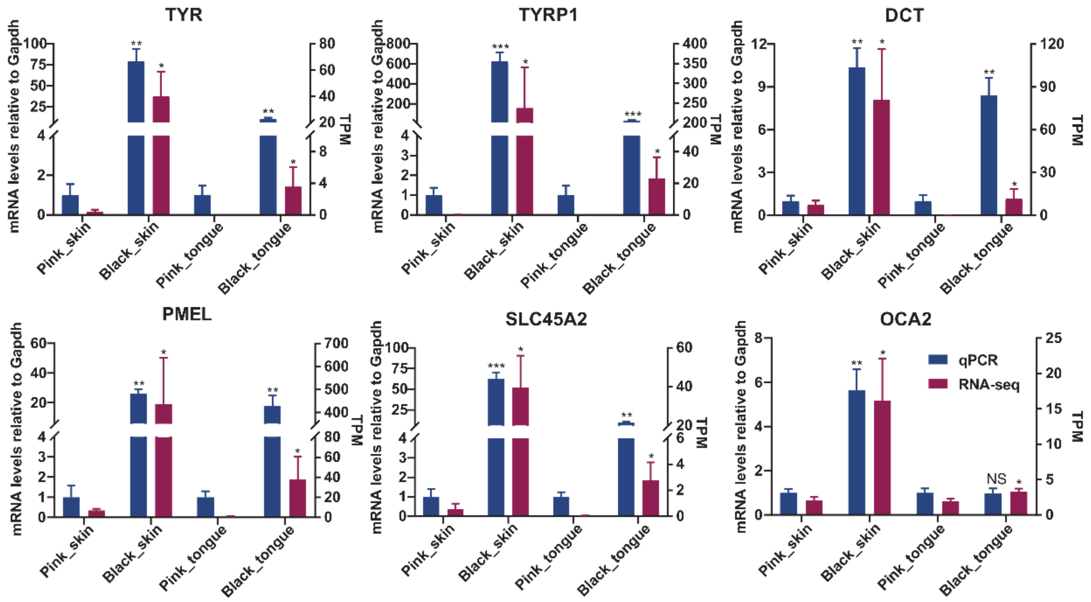

Figure S3. Differentially expressed genes qPCR experiment verification. The axis on the left represents expression levels of genes by qPCR, and the axis on the right represents transcripts Per Million (TPM) values of the genes by RNA-seq. Results are represented as the mean ± SEM, * $p < 0.05$, ** $p < 0.01$, *** $p < 0.001$, and NS indicates no significance.

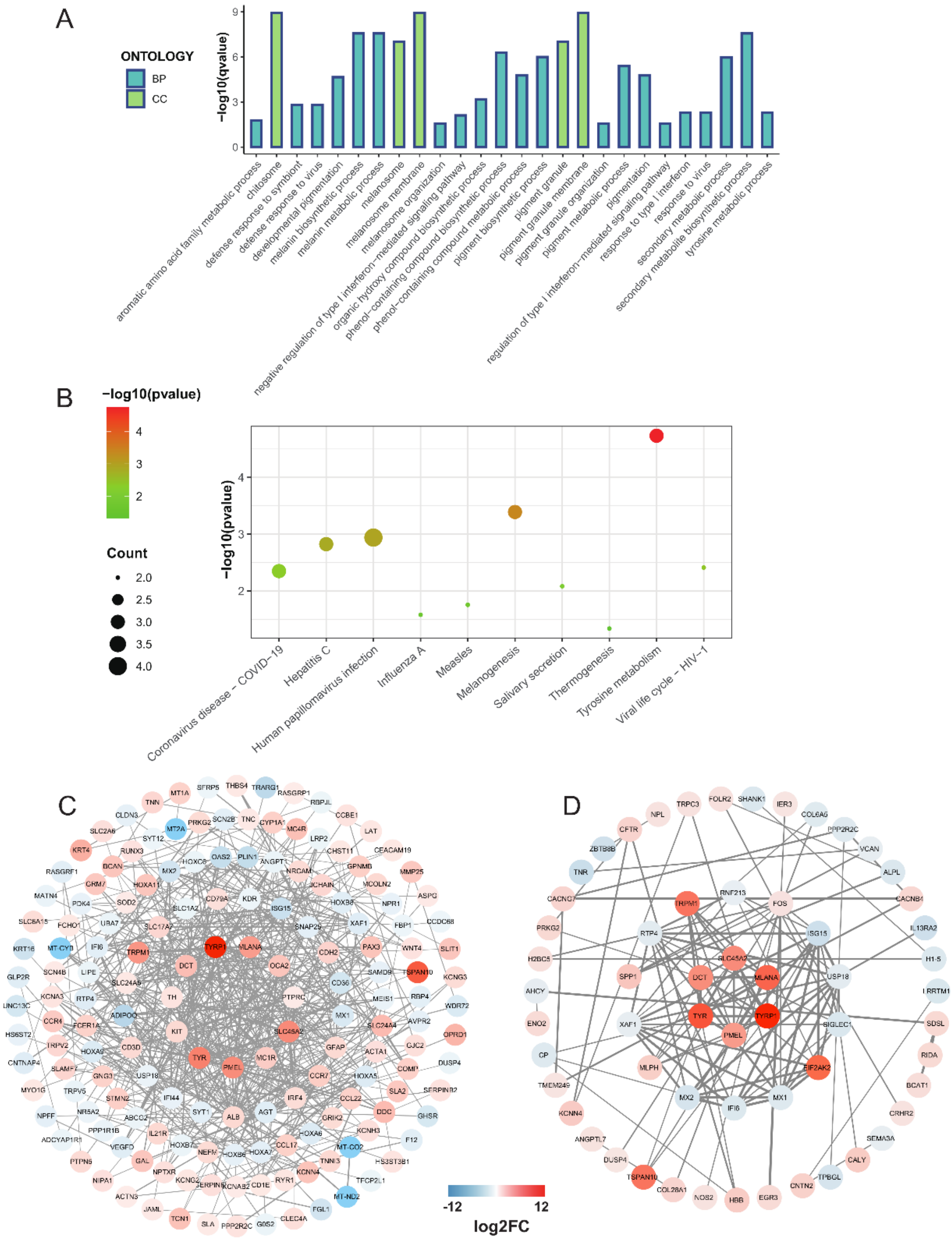

Figure S4. Genes function enrichment analysis and PPI networks. (A) GO enrichment analysis of shared differential genes in skin group and tongue group. (B) KEGG enrichment analysis of shared differential genes in the skin group and tongue group. (C) Black and pink skin differential gene PPI networks. (D) Black and pink tongue differential gene PPI networks.

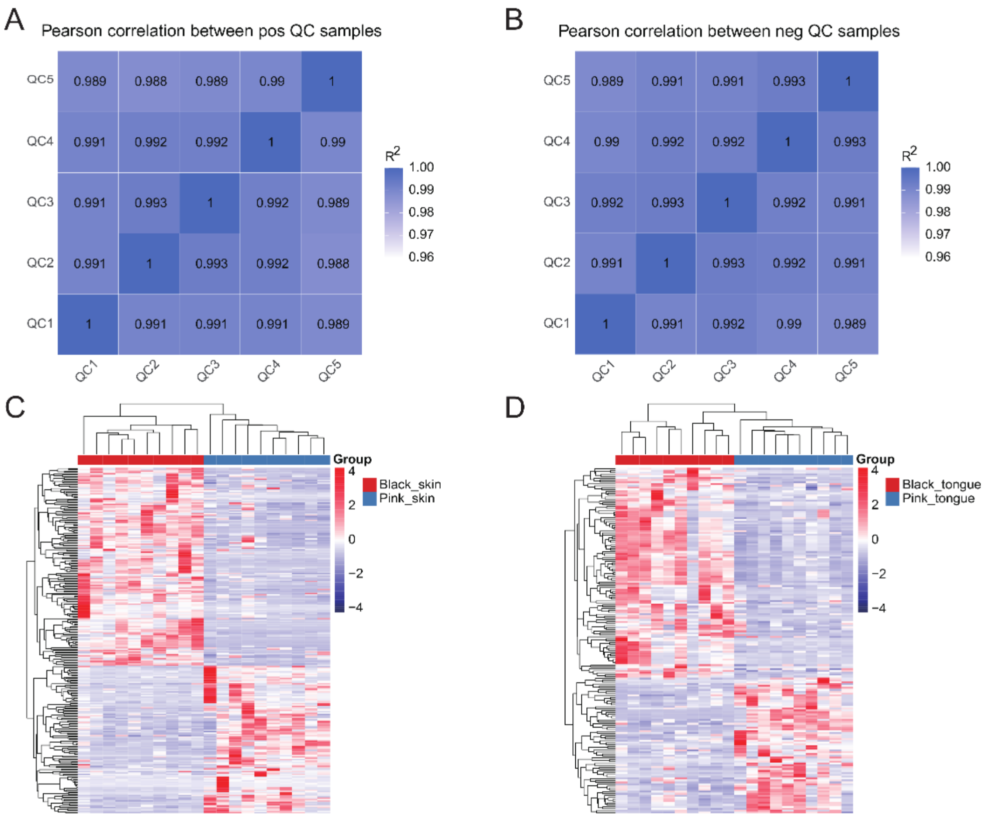

Figure S5. Metabolomic sample quality control and DEMs clustering. (A) Correlation plot between QC samples in positive ion mode. (B) Correlation plot between QC samples in negative ion mode. Heatmaps of Black_vs_Pink_skin (C) and Black_vs_Pink_tongue (D) DEMs clustering.
